# Bilateral Nonsyndromic Sensorineural Hearing Loss Caused by a NARS2 Mutation

**DOI:** 10.7759/cureus.31467

**Published:** 2022-11-14

**Authors:** Fawzia Al-Sharif, Hussain Alsadeq, Aahid Rozan, Molham B Halabi, Hamzah Badwilan, Adel A Mohammed, Moshiur Rahman, Turki Balgith

**Affiliations:** 1 Pediatrics, Saudi German Hospital Jeddah, Jeddah, SAU; 2 Medicine and Surgery, Batterjee Medical College, Jeddah, SAU

**Keywords:** mitochondrial disease, hearing loss, nonsyndromic deafness, aminoacyl-trna synthetase, nars2

## Abstract

Mitochondrial diseases disrupt the process of energy generation by the mitochondria, leading to manifestations that can affect almost any organ in the body. Although various possible clinical phenotypes can result, neurological and neuromuscular affection is most frequently encountered. NARS2 encodes an enzyme responsible for the conjugation of asparagine to its cognate mitochondrial transfer ribonucleic acid (tRNA) molecule, representing an essential step necessary for effective mitochondrial protein synthesis. As such, mutations in this gene can lead to poor mitochondrial gene expression and, consequently, poor energy output resulting in disease. Pathogenic variants in NARS2 have been known to cause neurodegenerative and myopathic syndromes in combined oxidative phosphorylation deficiency 24 (COXPD24). However, nonsyndromic autosomal recessive deafness 94 (DFNB94), with which only one family is known to be affected, has also been reported concerning NARS2. Our report demonstrates the association of a new pathogenic variant in mitochondrial asparaginyl-tRNA synthetase (NARS2) with nonsyndromic sensorineural hearing loss, thus confirming biallelic mutations in NARS2 as a cause of nonsyndromic deafness.

## Introduction

Mitochondrial diseases are a large and diverse group of disorders that affect the ability of mitochondria to generate energy, typically by oxidative phosphorylation. This process relies on a host of different mitochondrial and nuclear-encoded proteins responsible for the assembly and functioning of the electron transport chain. Genetic variants in any of these proteins can lead to mitochondrial dysfunction and disease [1]. Variants that encode mitochondrial aminoacyl-transfer ribonucleic acid (tRNA) synthetases (mt-aaRSs) have recently been linked to human disease. These mt-aaRSs are responsible for charging mitochondrial tRNA with their respective amino acids and thus play a key role in mitochondrial protein synthesis [2]. In 2007, Scheper et al. uncovered DARS2 mutations in patients with leukoencephalopathy with brain stem and spinal cord involvement and lactate elevation, marking the first known cases linking mt-aaRS mutations to disease in humans [3]. Since then, mutations in other mt-aaRS genes, including NARS2, have also been associated with mitochondrial disease.

The NARS2 gene (MIM: 612803), located on 11q14.1, codes for mitochondrial asparaginyl-tRNA synthetase 2. Like all mt-aaRSs, the enzyme is nuclear encoded and transported to the mitochondria. Its main function is to catalyze the aminoacylation of asparagine to tRNA molecules [4]. While NARS2 is widely expressed in human tissues, variants in the gene seem to preferentially affect tissues with high energy demand, such as the brain, cochlea, and muscle, similar to other mt-aaRS disorders [2]. Homozygous mutations in NARS2 have been associated with combined oxidative phosphorylation deficiency 24 (COXPD24; MIM: 616239) and autosomal recessive deafness 94 (DFNB94; MIM: 618434).

COXPD24 is an autosomal recessive mitochondrial disorder that often manifests early in life with seizures, hypotonia, myopathy, hearing impairment, and overall delay and/or regression of cognitive and motor development [5-7]. Computed tomography (CT) and magnetic resonance imaging (MRI), if performed, may reveal bilateral brain lesions or diffuse progressive cerebral atrophy indicative of Alpers or Leigh syndrome [8,9]. Autosomal recessive deafness 94 (DFNB94) is characterized by bilateral nonsyndromic sensorineural hearing loss [9]. Due to the limited number of cases involving NARS2 mutations, a correlation between genotype and phenotype has not yet been established, and the mechanisms behind NARS2-associated disease have not been extensively studied. We describe a patient with homozygous mutations in NARS2 who presented with a rarely seen phenotype of sensorineural hearing loss without other significant findings.

## Case presentation

A three-year-old Saudi boy presented to the clinic at age 14 months with a lack of response to sound, even loud noises, for several months. A pediatric evaluation revealed that language development was delayed compared to that expected for his age, likely due to hearing loss, but motor and cognitive developmental milestones were within the reference range. The patient was born at term by uncomplicated cesarean section to consanguineous healthy parents and was considered normal at birth. However, newborn hearing screening was not performed. He also had a positive family history of sensorineural hearing loss. Physical examination did not reveal abnormalities or deformities. The patient was seen and examined by an otolaryngologist and an audiologist. A normal tympanogram ruled out middle ear pathology. We tested otoacoustic emissions and cochlear microphonics, which were preserved, and auditory brainstem response testing revealed bilateral profound sensorineural hearing loss characteristic of auditory neuropathy. The patient was started on two behind-the-ear hearing aids with a low gain due to an initially mild type of hearing loss; his aided response was satisfactory.

After six months, the parents returned due to a lack of benefits from the patient's hearing aids. The results of his laboratory evaluation, including complete blood count and coagulation and biochemical tests, were within reference ranges. A head CT scan and MRI revealed no abnormalities in the middle ear, inner ear, skull, brain, or cerebellopontine angle bilaterally. One month later, he underwent cochlear implant surgery on his right ear and uses MED-EL SONNET 2 (MED-EL Medical Electronics, Innsbruck, Austria).

We suspected autosomal recessive disease and performed whole exome sequencing (WES) for the patient and his parents (Table 1). WES revealed a missense variant in the NARS2 gene (NARS2 c.506T>A; p.Phe169Tyr), with the patient being homozygous for the variant while his parents are heterozygous carriers. No other variants relevant to the patient's condition were found.

**Table 1:** Results of whole exome sequencing Abbreviations: HMZ, homozygous; HTZ, heterozygous; MAF, minor allele frequency; ACMG, American College of Medical Genetics and Genomics; CADD, Combined Annotation-Dependent Depletion; VUS, variant of uncertain significance. ^a^Based on gnomAD data ^b^CADD score predictions: (Deleterious: ≥20, Likely deleterious: 10-19, Unlikely deleterious: <10)

Gene (transcript)	cDNA change	Amino acid change	Impact	Zygosity			MAF^a^	ACMG Classification (CADD Score^b^)
				Patient	Mother	Father		
NARS2 (NM_024678.6)	c.506T>A	p.Phe169Tyr	Missense	HMZ	HTZ	HTZ	0.0004%	VUS (21.6)

## Discussion

In this case, we identified a homozygous NARS2 missense mutation: NARS2 c.506T>A; p.Phe169Tyr. This mutation is found in databases with extremely low frequency and has no previous record of any homozygote; it is located in the catalytic domain and affects a moderately conserved amino acid residue. In silico algorithms developed to predict the effect of missense variants show mixed findings. Some, including SIFT [10] and PolyPhen-2 [11], suggest the variant is tolerated or benign, while others, such as CADD [12] and MutationTaster [13], predict that the variant is damaging or disease-causing. This discrepancy could partially explain the less severe phenotype in this patient compared to other NARS2-associated diseases caused by unanimously "damaging" variants. According to the American College of Medical Genetics and Genomics classification [14], this mutation is considered a variant of uncertain significance at the time of writing.

Mitochondrial diseases are a large group of clinically heterogeneous disorders and can involve various organ systems [1]. Diseases specifically caused by mt-aaRS mutations, including most NARS2 variants, typically involve the central nervous system in the form of specific syndromes [2]. The first NARS2-associated disease was reported in 2015, describing a young boy with diffuse degeneration of cerebral gray matter diagnosed as Alpers syndrome [8]. Since then, more cases have emerged, adding to clinical variability and the list of pathogenic variants in NARS2-associated disease. It is now known that, in addition to Alpers syndrome, NARS2 mutations can present as Leigh syndrome, mitochondrial myopathy, hemiconvulsion-hemiplegia-epilepsy syndrome, developmental delay, epilepsy, and neonatal diabetes syndrome, and nonsyndromic hearing loss [5,6,9,15]. In the context of a pathogenic variant of NARS2, these presentations fall under the diagnosis of combined oxidative phosphorylation deficiency 24 (COXPD24); the exception is nonsyndromic hearing loss, which is its own entity.

Our patient presented with only bilateral prelingual sensorineural hearing loss without any other signs or symptoms of the central nervous system or muscle involvement (e.g., seizures, dementia, hypotonia, paralysis) that would typically be found in COXPD24. Furthermore, head and neck CT and MRI scans revealed no abnormal findings indicative of NARS2-associated syndromes such as Alpers or Leigh syndrome. Laboratory tests did not show significant findings and, apart from delayed language development, cognitive and motor development milestones were otherwise within reference ranges. This presentation resembles the only known report of nonsyndromic hearing loss occurring due to a NARS2 mutation, labeled autosomal recessive deafness 94 (DFNB94). In 2015, Simon et al. described a consanguineous family in which five adult members possessed a homozygous missense mutation and had nonsyndromic hearing loss without any other clinical characteristics, apart from possible premature menopause in two family members; thus, the designation of DFNB94 was given [9]. Other mt-aaRS mutations may also cause hearing loss, whether nonsyndromic or associated with specific syndromes. For example, mutations in the KARS gene can cause nonsyndromic autosomal recessive deafness 89 (DFNB89), while mutations in the LARS2 or HARS2 genes can cause Perrault syndrome, a disorder of sensorineural hearing loss and ovarian dysfunction [16-18].

An aspect to consider is the patient's age. At age three, it is difficult to predict whether more signs and symptoms will appear as the patient ages. Although sensorineural deafness can be the first sign of COXPD24, neurological and motor manifestations typically appear soon afterward [7,9]. However, due to the variable clinical presentation of NARS2-associated disease, early sensorineural deafness can be followed by further manifestations years later into childhood. Vafaee-Shahi et al. reported the cases of two siblings affected by the same missense mutation in NARS2 [19]. Both siblings were diagnosed with bilateral sensorineural hearing loss within the first 12 months of life, but one subsequently developed generalized seizures at age 14 months while the other developed the same at age seven years. Both were eventually diagnosed with COXPD24.

As such, in our case, a diagnosis of COXPD24 cannot be definitively ruled out. Although the patient's family history is positive for isolated sensorineural hearing loss that occurs in the absence of other symptoms, suggesting a diagnosis of DFNB94, follow-up of the patient is necessary to exclude other diagnoses reasonably.

## Conclusions

This report identifies a newly disease-causing variant in the NARS2 gene and validates NARS2 as a cause of early-onset nonsyndromic sensorineural hearing loss (DFNB94), adding to the genotypic and phenotypic variability of NARS2-associated disease. We suggest that using whole exome sequencing (including mt-aaRS genes) is beneficial in evaluating the patient with isolated sensorineural hearing loss by informing further management decisions and counseling for this condition. More studies are necessary to analyze the exact effects of NARS2 variants on mitochondrial protein synthesis, allowing the development of a more targeted approach to managing the condition.

## References

[1] Boczonadi V, Horvath R (2014). Mitochondria: impaired mitochondrial translation in human disease. Int J Biochem Cell Biol.

[2] Fine AS, Nemeth CL, Kaufman ML, Fatemi A (2019). Mitochondrial aminoacyl-tRNA synthetase disorders: an emerging group of developmental disorders of myelination. J Neurodev Disord.

[3] Scheper GC, van der Klok T, van Andel RJ (2007). Mitochondrial aspartyl-tRNA synthetase deficiency causes leukoencephalopathy with brain stem and spinal cord involvement and lactate elevation. Nat Genet.

[4] Sofou K, Kollberg G, Hedberg-Oldfors C, Oldfors A (2021). The phenotypic variability and natural history of NARS2 associated disease. Eur J Paediatr Neurol.

[5] Vanlander AV, Menten B, Smet J (2015). Two siblings with homozygous pathogenic splice-site variant in mitochondrial asparaginyl-tRNA synthetase (NARS2). Hum Mutat.

[6] Mizuguchi T, Nakashima M, Kato M (2017). PARS2 and NARS2 mutations in infantile-onset neurodegenerative disorder. J Hum Genet.

[7] Seaver LH, DeRoos S, Andersen NJ (2018). Lethal NARS2-related disorder associated with rapidly progressive intractable epilepsy and global brain atrophy. Pediatr Neurol.

[8] Sofou K, Kollberg G, Holmström M (2015). Whole exome sequencing reveals mutations in NARS2 and PARS2, encoding the mitochondrial asparaginyl-tRNA synthetase and prolyl-tRNA synthetase, in patients with Alpers syndrome. Mol Genet Genomic Med.

[9] Simon M, Richard EM, Wang X (2015). Mutations of human NARS2, encoding the mitochondrial asparaginyl-tRNA synthetase, cause nonsyndromic deafness and Leigh syndrome. PLoS Genet.

[10] Kumar P, Henikoff S, Ng PC (2009). Predicting the effects of coding non-synonymous variants on protein function using the SIFT algorithm. Nat Protoc.

[11] Adzhubei IA, Schmidt S, Peshkin L (2010). A method and server for predicting damaging missense mutations. Nat Methods.

[12] Rentzsch P, Witten D, Cooper GM, Shendure J, Kircher M (2019). CADD: predicting the deleteriousness of variants throughout the human genome. Nucleic Acids Res.

[13] Schwarz JM, Rödelsperger C, Schuelke M, Seelow D (2010). MutationTaster evaluates disease-causing potential of sequence alterations. Nat Methods.

[14] Richards S, Aziz N, Bale S (2015). Standards and guidelines for the interpretation of sequence variants: a joint consensus recommendation of the American College of Medical Genetics and Genomics and the Association for Molecular Pathology. Genet Med.

[15] Yagasaki H, Sano F, Narusawa H (2022). Compound heterozygous variants of the NARS2 gene in siblings with developmental delay, epilepsy, and neonatal diabetes syndrome. Am J Med Genet A.

[16] Santos-Cortez RL, Lee K, Azeem Z (2013). Mutations in KARS, encoding lysyl-tRNA synthetase, cause autosomal-recessive nonsyndromic hearing impairment DFNB89. Am J Hum Genet.

[17] Pierce SB, Gersak K, Michaelson-Cohen R (2013). Mutations in LARS2, encoding mitochondrial leucyl-tRNA synthetase, lead to premature ovarian failure and hearing loss in Perrault syndrome. Am J Hum Genet.

[18] Pierce SB, Chisholm KM, Lynch ED (2011). Mutations in mitochondrial histidyl tRNA synthetase HARS2 cause ovarian dysgenesis and sensorineural hearing loss of Perrault syndrome. Proc Natl Acad Sci U S A.

[19] Vafaee-Shahi M, Farhadi M, Razmara E (2022). Novel phenotype and genotype spectrum of NARS2 and literature review of previous mutations. Ir J Med Sci.

